# Digital Cephalometric Analysis: Unveiling the Role and Reliability of Semi-automated OneCeph, Artificial Intelligence-Powered WebCeph Mobile App, and Semi-automated Computer-Aided NemoCeph Software in Orthodontic Practice

**DOI:** 10.7759/cureus.72948

**Published:** 2024-11-03

**Authors:** Alisha Chuchra, Kimmi Gupta, Reetu Arora, Shweta Bindra, Nupur Hingad, Amit Babbar

**Affiliations:** 1 Orthodontics, Adesh Institute of Dental Sciences and Research, Bathinda, IND; 2 Prosthodontics, Adesh Institute of Dental Sciences and Research, Bathinda, IND; 3 Conservative Dentistry and Endodontics, Adesh Institute of Dental Sciences and Research, Bathinda, IND; 4 Prosthodontics, Sri Sukhmani Dental College and Hospital, Dera Bassi, IND; 5 Oral and Maxillofacial Pathology, Sri Sukhmani Dental College and Hospital, Dera Bassi, IND

**Keywords:** cephalometric analysis, icc, nemoceph, oneceph, reliability, tracing, webceph

## Abstract

Background

Cephalometric analysis is essential in orthodontic diagnosis and treatment planning. With the emergence of digital tools for cephalometric analysis such as OneCeph, WebCeph, and NemoCeph, there is growing interest in their reliability compared to traditional manual tracings. This study aimed to compare the reliability of these digital tools with manual tracings in doing cephalometric analysis.

Methodology

Cephalometric radiographs from a diverse patient population were analyzed using OneCeph (NXS, Hyderabad, India), WebCeph (AssembleCircle Corp., Republic of Korea), NemoCeph (Nemotec, Madrid, Spain), and manual tracings by experienced orthodontists. Interobserver reliability and agreement with manual tracings were assessed using the intraclass correlation coefficient (ICC).

Results

The comparison of cephalometric measurements using the four methods - manual, OneCeph, WebCeph, and NemoCeph - revealed significant differences in the Sella-Nasion to Point A angle (SNA) (P = 0.002) and angle of difference between Sella-Nasion to Point A angle and Sella-Nasion to Point B angle (ANB) (P<0.001). Specifically, WebCeph produced significantly higher SNA measurements than manual tracing, while NemoCeph, OneCeph, and WebCeph yielded higher ANB measurements than manual tracing. There were no significant differences in other measurements, including Sella-Nasion to Point B angle (SNB), Nasion to Point A (N to Pt A), Nasion to Point B (N to Pt B), Gonion-Gnathion to Sella-Nasion angle (Go-Gn to SN), Lower Anterior Facial Height (LAFH), Y-axis (growth axis angle), facial axis, the sum of posterior measurements, and various angular and linear distances [1 to NA, 1 to SN, 1 to NB, 1 to Apog, Incisor Mandibular Plane Angle (IMPA), Sella to Upper Lip (S to UL), and Sella to Lower Lip (S to LL)]. The reliability analysis indicated a strong internal consistency with Cronbach’s α values of 0.811 for manual vs. NemoCeph, 0.859 for manual vs. OneCeph, and 0.861 for manual vs. WebCeph, and good agreement in the ICC (P<0.001).

Conclusion

OneCeph, WebCeph, and NemoCeph demonstrate promising reliability for cephalometric analysis. However, the results should be interpreted with caution, considering the limitations of digital tools. Ongoing research and collaboration among developers, researchers, and clinicians are essential to validate these the performance of these tools and improve their clinical applicability.

## Introduction

In the realm of orthodontics, cephalometric analysis plays a vital role in treatment planning, diagnosis, and assessment of treatment outcomes. In recent years, the field of orthodontics has witnessed a significant shift towards digital technologies for cephalometric analysis. Recent developments in diagnostic and treatment technologies utilizing computer-based systems enable practitioners to examine issues from multiple perspectives, facilitating precise diagnosis and treatment strategies. Traditionally, cephalometric tracings were performed manually, requiring intricate hand-eye coordination, meticulous attention to detail, and a considerable time investment. However, with the advent of digital imaging and software advancements such as Dolphin Imaging, NemoCeph, Vistadent, and various mobile applications such as OneCeph, WebCeph, and CephNinja, the landscape of cephalometric analysis has experienced a major shift from traditional tracing to sophisticated computer-enhanced digital cephalometric assessment systems [1]. These digital tools offer a more automated and standardized approach to cephalometric analysis, leveraging advanced algorithms and image processing techniques to streamline the identification and measurement of landmarks [2,3].

A recent study by Chen et al. [4] found no notable discrepancies between traditional and computer-enhanced cephalometric assessment, with deviations in one or two parameters. But nowadays, with the current trend of increased smartphone usage, mobile applications have assumed the function of software programs in performing cephalometric evaluations. Mobile apps running on Android or other operating systems can either be fully automated (driven by artificial intelligence) or semi-automated (necessitating manual landmark identification). A recent study by Mohan et al. [5] found the OneCeph mobile app to be reliable except in one or two parameters. Another study by Yassir et al. [6] compared the WebCeph computer program with the AutoCAD software and found significant differences in measured parameters. Since digital cephalometrics is still being explored and is new to orthodontists, the accuracy and reliability of these methods remain in question. The available research on the dependability of smartphone applications remains insufficient, and currently, there are no studies comparing the reliability of semi-automatic, artificial intelligence-powered smartphone apps with standard computer-assisted software and traditional manual tracings. Hence, this study was done with the aim of comparing the reliability of WebCeph (artificial intelligence), OneCeph (semi-automatic Android app), and NemoCeph (standard computer-assisted software) with manual tracing by conventional methods, to provide orthodontists with an ergonomic application that helps in evidence-based decision-making for initiating and proceeding with orthodontic treatment at the earliest.

## Materials and methods

Study design and setting

Three digital tools, OneCeph (NXS, Hyderabad, India), WebCeph (AssembleCircle Corp., Republic of Korea), and NemoCeph (Nemotec, Madrid, Spain) were selected based on their popularity, availability, and features relevant to cephalometric analysis. Both OneCeph and WebCeph are mobile applications whereas NemoCeph is a computer software. The study was conducted on 130 cephalometric radiographs obtained from the patient records of the Department of Orthodontics, Adesh Institute of Dental Sciences and Research, Bathinda, after the approval by the institutional ethical committee (AU/EC_BHW2K23/379) dated 24th January 2023. Hence, the study was undertaken between 1st February 2023 and 31st January 2024. 

Selection Criteria

The sample size was calculated using nMaster 2.0 software (Department of Biostatistics, Christian Medical College, Vellore, India), employing an equal allocation approach based on mean values derived from prior research [7]. The inclusion criteria for radiographs include a diverse range of patients with varying craniofacial morphologies and ages, ensuring a representative sample for analysis. Also, the cephalometric images were anonymized to protect patient confidentiality and comply with ethical standards.

Data sources and variables

Manual cephalometric tracings were performed independently by two experienced orthodontists. Landmark identification and measurements were conducted using traditional tracing methods with transparent overlays. Table 1 summarizes the angular and linear measurements used in the analysis, including key variables like Sella-Nasion to Point A angle (SNA), Sella-Nasion to Point B angle (SNB), angle of difference between SNA and SNB (ANB), and Incisor Mandibular Plane Angle (IMPA). Interobserver reliability for manual tracings was assessed by calculating intraclass correlation coefficients (ICCs) for selected landmarks and composite analysis measurements. The results demonstrated high ICC values (ICC>0.800), indicating strong agreement between the two orthodontists for manual landmark identification. Cephalometric images were imported into OneCeph, WebCeph, and NemoCeph for digital analysis. Each tool's respective algorithms and user interfaces were utilized for landmark identification and measurements, ensuring consistency in the evaluation process. The same orthodontists also conducted the digital analysis to maintain uniformity across manual and digital methods. The ICC values for the digital tools varied, with OneCeph, WebCeph, and NemoCeph showing an ICC of >0.819, >0.800, and >0.912, respectively for both landmark identification and composite analysis measurements. These values reflect varying levels of agreement between the digital tools and manual tracings, as illustrated in Table 1.

**Table 1:** Composite cephalometric analysis

Angular Measurements	Linear Measurements
SNA (Sella-Nasion-Point A angle)	N to Pt A (Nasion to Point A)
SNB (Sella-Nasion-Point B angle)	N to Pt B (Nasion to Point B)
ANB (Difference Between SNA and SNB angles)	LAFH (Lower Anterior Facial Height)
IMPA (Incisor Mandibular Plane Angle)	1 to NA (Incisor to Nasion-A Line)
Go-Gn to SN (Gonion-Gnathion to Sella-Nasion Angle)	1 to NB (Incisor to Nasion-B Line)
Y-Axis (Vertical Axis Angle)	1 to SN (Incisor to Sella-Nasion Line)
1 to NA (Incisor to Nasion-A Line)	1 to Apog (Incisor to Apogee)
Facial Axis (Angle of Facial Axis)	S to UL (Sella to Upper Lip)
Sum of Posterior Angle	S to LL (Sella to Lower Lip)

Statistical Analysis

Information on linear and angular measurements from different tracing techniques was documented using Microsoft Excel (Microsoft Corporation, Redmond, Washington, United States) and evaluated with IBM SPSS Statistics for Windows, Version 20 (Released 2013; IBM Corp., Armonk, New York, United States). Descriptive statistics were calculated as mean and standard deviation. The comparison of cephalometric parameters among various methods was done using analysis of variance (ANOVA) followed by Tukey’s post-hoc test for pair-wise comparisons. The level of significance for the present study was fixed at a P-value of less than 0.05.

## Results

Table 2 illustrates the comparison of cephalometric measurements obtained through four different methods: manual, OneCeph, WebCeph, and NemoCeph. The analysis revealed statistically significant differences in the measurement of SNA (P = 0.002) and ANB (P < 0.001) across the various methods. However, no statistically significant differences were observed in the other measurements.

**Table 2:** Comparison of cephalometric measurements obtained by various methods Statistically significant (P<0.05, ANOVA applied), SNA: Sella-Nasion to Point A angle, SNB: Sella-Nasion to Point B angle, ANB: Difference between SNA and SNB angles, N to Pt A: Nasion to Point A linear distance, N to Pt B: Nasion to Point B linear distance, Go-Gn to SN: Gonion-Gnathion to Sella-Nasion angle, LAFH: Lower Anterior Facial Height, Y-axis: Growth axis angle, Sum of post: sum of posterior angles, 1 to NA (Angular): Angle between upper incisor and NA line, 1 to NA (Linear): Distance between upper incisor and NA line, 1 to SN: Upper incisor to Sella-Nasion line, 1 to NB (Angular): Angle between lower incisor and NB line, 1 to NB (Linear): Distance between lower incisor and NB line, 1 to Apog: Lower incisor to Apogee point distance, IMPA: Incisor Mandibular Plane Angle, S to UL: Sella to Upper Lip distance, S to LL: Sella to Lower Lip distance. Software: NemoCeph (Nemotec, Madrid, Spain), OneCeph (NXS, Hyderabad, India), and WebCeph (AssembleCircle Corp., Republic of Korea).

	Manual	NemoCeph	OneCeph	WebCeph	F-value	P-value
SNA	80.39±2.51	80.86±2.14	80.95±2.12	81.50±2.21	5.004	0.002
SNB	77.80±2.21	77.74±2.17	77.75±2.16	77.78±2.11	0.021	0.996
ANB	2.61±1.41	3.15±1.37	3.20±1.39	3.71±1.39	12.979	<0.001
N to pt A	2.25±1.66	2.27±1.65	2.27±1.65	2.29±1.63	0.018	0.997
N to pt B	7.34±2.66	7.22±2.54	7.22±2.54	7.51±2.51	0.356	0.785
Go-Gn to SN	30.85±7.18	32.63±20.00	32.71±19.97	32.79±19.96	0.346	0.792
LAFH	74.78±11.94	74.80±11.92	74.82±11.92	74.66±12.36	0.005	1.000
Y-axis	65.48±4.98	65.52±4.98	65.53±4.99	65.17±7.30	0.112	0.953
Facial axis	0.49±5.61	0.47±5.63	0.48±5.64	0.49±5.70	0.000	1.000
Sum of post	390.06±8.70	390.07±8.69	390.08±8.68	390.17±8.67	0.004	1.000
1 to NA (Angular)	31.50±7.64	31.51±7.63	31.52±7.64	31.58±7.63	0.003	1.000
1 to NA (Linear)	9.54±5.73	9.57±5.72	9.59±5.72	9.65±5.72	0.008	0.999
1 to SN	113.48±12.57	113.50±12.56	113.53±12.56	112.79±14.61	0.092	0.964
1 to NB (Angular)	28.92±8.60	28.68±8.82	28.69±8.82	28.85±8.79	0.023	0.995
1 to NB (Linear)	7.47±3.84	7.50±3.86	7.51±3.86	7.58±3.87	0.019	0.996
1 to Apog	3.12±3.69	3.18±3.71	3.19±3.72	3.26±3.75	0.027	0.994
IMPA	98.37±8.43	104.37±57.97	104.38±57.98	104.25±58.05	0.434	0.729
S to UL	0.99±2.57	1.03±2.60	1.04±2.61	1.11±2.71	0.044	0.988
S to LL	1.55±3.12	0.94±3.93	0.95±3.93	0.97±3.99	0.784	0.503

Table 3 shows the pairwise comparisons among the various methods of cephalometric measurements, revealing a statistically significant difference in the measurement of SNA between the manual method and WebCeph (P=0.001). The mean SNA measurement using WebCeph was significantly higher than that obtained using the manual method, while other differences in SNA measurements were not statistically significant. For ANB, statistically significant differences were observed between the manual and NemoCeph (P=0.013), manual and OneCeph (P=0.005), and manual and WebCeph (P<0.001) methods. The mean ANB measurements obtained with NemoCeph, OneCeph, and WebCeph were significantly higher than those obtained using the manual method. Higher mean ANB measurements were observed with WebCeph and were significantly different from NemoCeph (P=0.008) and OneCeph (P=0.022). No significant difference was observed between NemoCeph and OneCeph (P=0.989) in ANB measurement though.

**Table 3:** Pair-wise comparisons between the different methods Statistically significant (P<0.05, Tukey’s post-hoc test applied), SNA: Sella-Nasion to Point A, SNB: Sella-Nasion to Point B, ANB: Difference between SNA and SNB angles, n to pt A: Nasion to Point A, N to pt B: Nasion to Point B, Go Gn to SN: Gonion-Gnathion to Sella-Nasion, Sum of post: sum of posterior angles, LAFH: Lower Anterior Facial Height, sum of post: Sum of Posterior Measurements, 1 to NA: Incisor to Nasion-A, 1 to SN: Incisor to Sella-Nasion, 1 to NB: Incisor to Nasion-B, 1 to Apg: Incisor to Apex of the Goniion, IMPA: Incisor Mandibular Plane Angle, S to UL: Sella to Upper Lip, S to LL: Sella to Lower Lip Software: NemoCeph (Nemotec, Madrid, Spain), OneCeph (NXS, Hyderabad, India), and WebCeph (AssembleCircle Corp., Republic of Korea).

	Manual vs NemoCeph (P-value)	Manual vs OneCeph (P-value)	Manual vs WebCeph (P-value)	NemoCeph vs OneCeph (P-value)	NemoCeph vs WebCeph (P-value)	OneCeph vs WebCeph (P-value)
SNA	0.361	0.212	0.001	0.990	0.118	0.233
SNB	0.995	0.998	1.000	1.000	0.999	1.000
ANB	0.013	0.005	<0.001	0.989	0.008	0.022
N to pt A	0.999	0.999	0.996	1.000	0.999	0.999
N to pt B	0.982	0.982	0.954	1.000	0.809	0.809
Go Gn to SN	0.857	0.840	0.823	1.000	1.000	1.000
LAFH	1.000	1.000	1.000	1.000	1.000	1.000
Y-axis	1.000	1.000	0.973	1.000	0.963	0.960
Facial axis	1.000	1.000	1.000	1.000	1.000	1.000
Sum of post	1.000	1.000	1.000	1.000	1.000	1.000
1 to NA	1.000	1.000	1.000	1.000	1.000	1.000
1 to NA	1.000	1.000	0.999	1.000	1.000	1.000
1 to SN	1.000	1.000	0.976	1.000	0.974	0.971
1 to NB	0.996	0.997	1.000	1.000	0.999	0.999
1 to NB	1.000	1.000	0.996	1.000	0.998	0.999
1 to Apg	0.999	0.999	0.992	1.000	0.998	0.999
IMPA	0.785	0.784	0.795	1.000	1.000	1.000
S to UL	0.999	0.999	0.984	1.000	0.995	0.997
S to LL	0.577	0.586	0.621	1.000	1.000	1.000

The reliability statistics showed good internal consistency with a Cronbach’s α value of 0.811 (manual tracing vs NemoCeph), 0.859 (manual tracing vs OneCeph), and 0.861 (manual vs WebCeph) as shown in Table 4. The ICC also showed good agreement (P<0.001) with all the three groups.

**Table 4:** Reliability statistics Software: NemoCeph (Nemotec, Madrid, Spain), OneCeph (NXS, Hyderabad, India), and WebCeph (AssembleCircle Corp., Republic of Korea).

	Group	Cronbach’s α Value	Interclass Correlation (ICC) Value	p-value
Manual Tracing	NemoCeph	0.811	0.811	< 0.001
Manual Tracing	OneCeph	0.859	0.859	< 0.001
Manual tracing	WebCeph	0.861	0.861	< 0.001

## Discussion

Machine learning techniques have dramatically transformed diagnosis and treatment planning across all dental disciplines, with their influence in orthodontics increasing notably in recent years [2]. Mobile phone applications have given orthodontics a new life and have taken it a step further. Intrigued by the idea of using smartphones for cephalometric analysis, mobile-based cephalometric applications were introduced to make such analyses a pocket-friendly activity. Subsequently, softwares for cephalometric analysis were developed as applications on smartphones. However, there is a lack of published evidence comparing the gold standard manual tracing with computer software tracing, semi-automated tracing, and fully automated artificial intelligence-powered tracing programs of mobile apps in terms of reliability. Therefore, this research was carried out to compare the reliability of OneCeph (semi-automatic), WebCeph (artificial intelligence) Android app, and NemoCeph (computer-aided tracing program) with conventional manual tracing.

The results of the present study revealed varying levels of agreement between manual and digital cephalometric analyses across the three digital tools used. OneCeph, WebCeph, and NemoCeph showed ICC agreement of >0.819, >0.800, and >0.912, respectively for landmark identification and composite analysis measurements, which was in concordance with the study by Erkan et al. [8] who suggested that when evaluating software reliability, errors within the same examiner should be measured as they are much less than the interexaminer error. In their research, all intraexaminer correlation coefficients for repeated measurements were 0.90 or higher, with the exception of maxillary length (ANS-PNS), which was omitted from this study [8].

The consistency (comparison between digital and traditional methods) revealed statistically notable discrepancies among the various methods in the measurement of SNA (P=0.002) and ANB (P<0.001). There were no statistically significant differences in the measurement of other parameters used in the study. The mean SNA measurement using WebCeph was significantly higher than that obtained using the manual method. Also, the mean ANB measurements obtained with NemoCeph, OneCeph, and WebCeph were significantly higher than those obtained using the manual method. Webceph also showed statistically significant differences from NemoCeph (P=0.008) and OneCeph (P=0.022), which were in concordance with the study by Al Barakati et al. [9] who identified notable variances in SNA readings. Previous research suggests that it can be challenging to pinpoint the nasion accurately when the nasofrontal suture is not distinctly visible. Furthermore, the A point is situated on a curve, which may introduce minor measurement errors [10,11]. The inconsistency between conventional and digital measurements might also stem from variations in how the operator identifies certain cephalometric points on a mobile touchscreen, despite consistent results within each method [12]. Also, the algorithms and measurement techniques employed by OneCeph, WebCeph, and NemoCeph, and the user proficiency and familiarity with these software interfaces may influence the reliability [13]. Despite these limitations, it is crucial to highlight that the discrepancies in the values were under 1 mm or 0.5 degrees, which might be considered clinically insignificant. Therefore, the study provides valuable insights into the comparative reliability of mobile apps and software platforms with manual tracings in cephalometric analysis. While digital tools offer convenience and efficiency, their reliability compared to manual tracings varies and should be interpreted with caution in clinical practice [14,15].

Limitations of the study

The study's sample size of 130 cephalometric radiographs may not fully represent all craniofacial variations, potentially limiting the generalization of the findings. The comparison focused on only three digital tools (OneCeph, WebCeph, and NemoCeph), and the results might not apply to other software. Variability in landmark identification and measurement accuracy among tools could introduce bias, and differences in the operator skill were not considered. Although statistically significant, the measurement differences were often below 1 mm or 0.5 degrees, suggesting limited clinical impact. Additionally, using manual tracings as the gold standard introduces potential variability due to human error.

## Conclusions

The reliability of OneCeph, WebCeph, and NemoCeph in performing cephalometric analysis shows promise; however, caution is warranted when interpreting the results. Orthodontic practitioners should carefully weigh the strengths and limitations of these digital tools alongside their clinical expertise to ensure accurate and effective treatment planning. Continued research is necessary to validate the reliability of these tools across diverse patient populations and clinical settings. Furthermore, collaboration between software developers, orthodontic researchers, and clinicians is crucial to address the limitations of existing digital tools and enhance their functionality and usability. Incorporating feedback from end users into software updates is essential to ensure that these tools align with clinical needs and preferences.
